# TM4SF1 promotes the self-renewal of esophageal cancer stem-like cells and is regulated by miR-141

**DOI:** 10.18632/oncotarget.13866

**Published:** 2016-12-10

**Authors:** Lei Xue, Xiying Yu, Xingran Jiang, Xin Deng, Linlin Mao, Liping Guo, Jinhu Fan, Qinqxia Fan, Liuxing Wang, Shih-Hsin Lu

**Affiliations:** ^1^ Department of Oncology, the First Affiliated Hospital of Zhengzhou University, Zhengzhou, China; ^2^ Department of Etiology and Carcinogenesis and State Key Laboratory of Molecular Oncology, National Cancer Center/Cancer Hospital, Chinese Academy of Medical Sciences (CAMS) & Peking Union Medical College (PUMC), Beijing, China; ^3^ Beijing Key Laboratory for Carcinogenesis and Cancer Prevention, Beijing, China; ^4^ Department of Cancer Epidemiology, National Cancer Center/Cancer Hospital, Chinese Academy of Medical Sciences (CAMS) & Peking Union Medical College (PUMC), Beijing, China; ^5^ Current address: Department of Pathology, Beijing ChaoYang Hospital, Capital Medical University, Beijing, China

**Keywords:** cancer stem-like cells, TM4SF1, miR-141, esophageal cancer, ESCC

## Abstract

Cancer stem-like cells have been identified in primary human tumors and cancer cell lines. Previously we found TM4SF1 gene was highly expressed in side population (SP) cells from esophageal squamous cell carcinoma (ESCC) cell lines, but the role and underlying mechanism of TM4SF1 in ESCC remain unclear. In this study, we observed TM4SF1 was up-regulated but miR-141 was down-regulated in SP cells isolated from ESCC cell lines. TM4SF1 could stimulate the self-renewal ability and carcinogenicity of esophageal cancer stem-like cells, and promote cell invasion and migration. In miR-141 overexpression cells, the expression of TM4SF1 was significantly reduced. We also found that overexpression of miR-141 could abolish the self-renewal ability and carcinogenicity of esophageal cancer stem-like cells and decrease cell invasion and migration by suppressing TM4SF1. Consequently, TM4SF1 is a direct target gene of miR-141. The regulation of TM4SF1 by miR-141 may play an important role in controlling self-renewals of esophageal cancer stem-like cells. It may also promote the development of new therapeutic strategies and efficient drugs to target ESCC stem-like cells.

## INTRODUCTION

Esophageal cancers rank as the 8th most common cancer and the 4th highest cause of cancer-related mortality in the world [1]. Esophageal squamous cell carcinoma (ESCC) is the most common histological subtype and accounts for almost 90% of all esophageal cancers in China [2]. Data from 31 urban cancer registration areas in China in 2015 showed that ESCC is the 3rd leading cause of cancer morbidities and 4th cancer mortalities [3]. The recurrence and metastasis rates of ESCC are very high after surgical treatment and the prognosis is usually poor [4]. The main reason of recurrence and metastasis is likely due to the residual malignant cells in tumor with stem-cell-like potential. These tiny amount cells are called as “tumor-initiating cells”, “cancer stem cells”,or “cancer stem-like cells”.

Cancer stem-like cells were first identified in hematological malignancies, mainly in acute myelogenous leukemia [5]. Then cancer stem-like cells were found in solid tumors [6–10]. In the absence of known surface antigens of ESCC cancer stem-like cells, previously we effectively isolated esophageal cancer stem-like cells using side population (SP) sorting with Hoechst 33342 [11]. Our results showed that SP cells share certain common features of cancer stem-like cells, such as the ability of self-renewal, highly proliferation, carcinogenicity and drug resistance [11–13].

TM4SF1 is a small protein which has four-transmembrane-domain that also known as L6-Ag. Studies have reported that TM4SF1 is over-expressed in many malignancies [14–18], but the expression profiles of TM4SF1 in esophageal cancer remains unclear. It is also highly expressed in the endothelial cells of angiogenic blood vessels that supplied human cancers [19]. Besides, TM4SF1 was suggested as a possible marker of stem-like cells in thyroid cancer cells and serve as a surface protein marker which singly identifies mesenchymal stem cells from diverse cell sources, in particular, fibroblast-rich connective tissues [20, 21].

MicroRNAs (miRNAs) can function as either tumor suppressors or oncogenes by regulating their down-stream target genes [22]. MiR-141 is a member of the miR-200 family, and is reported to be up or down regulated in many type of cancers [23–33]. The downregulation of miR-141 suggests that normal and breast cancer stem cells share common molecular mechanisms that regulate stem cell functions such as self-renewal, proliferation and EMT [33]. However, the expression pattern of miR-141 and its role in ESCC and cancer stem-like of ESCC are poorly understood.

In previous study, we sorted SP and NSP cells and used gene microarray to indentify stem cell-related genes and miRNAs which were including TM4SF1 and miR-141. In this study, we investigated the role of TM4SF1 and miR-141 in cancer stem-like cells. Our results indicated TM4SF1 was a direct target gene of miR-141. TM4SF1 and miR-141 together played important roles in maintaining the self-renewal ability and carcinogenicity of esophageal cancer stem-like cells.

## RESULTS

### TM4SF1 and miR-141 are inversely expressed in esophageal cancer stem-like cells

**Figure 1:**
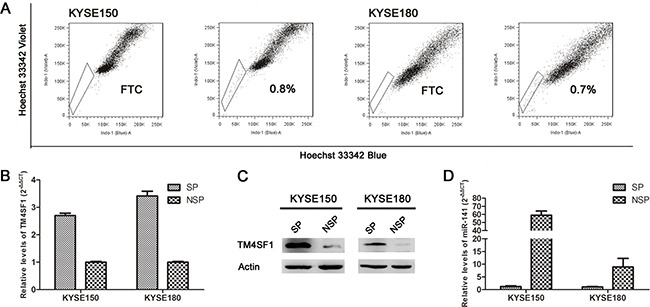
The expression of TM4SF1 and miR-141 in esophageal cancer stem-like cells **A**. Side population (SP) analysis of KYSE150 and KYSE180 cell lines. Incubation with 5pM FTC abolished the SP cell fraction. **B, C**. In SP and non-SP cells of KYSE150 and KYSE180 cell lines, TM4SF1 expression level was measured by Western blot and real-time PCR, and **D**. miR-141 expression level was measured by real-time PCR.

Previously, the result from gene-array demonstrates that TM4SF1 and miR-141 were inversely expressed in SP and none-SP cells. In this study, we did SP analysis to sorted SP and none-SP cells in esophageal cancer cell lines KYSE150 and KYSE180 (Figure 1A). The expression of TM4SF1 was measured by real-time PCR and Western blot. As shown in Figure 1B and 1C, TM4SF1 expression was up-regluated in SP cells which were consistent with the gene-array. Concurrently, we found miR-141 expression was down-regulated in SP cells by real-time PCR (Figure 1D). These results demonstrated that TM4SF1 and miR-141 may play an important role in esophageal cancer stem-like cells.

### TM4SF1 regulates the SP phenotype

**Figure 2:**
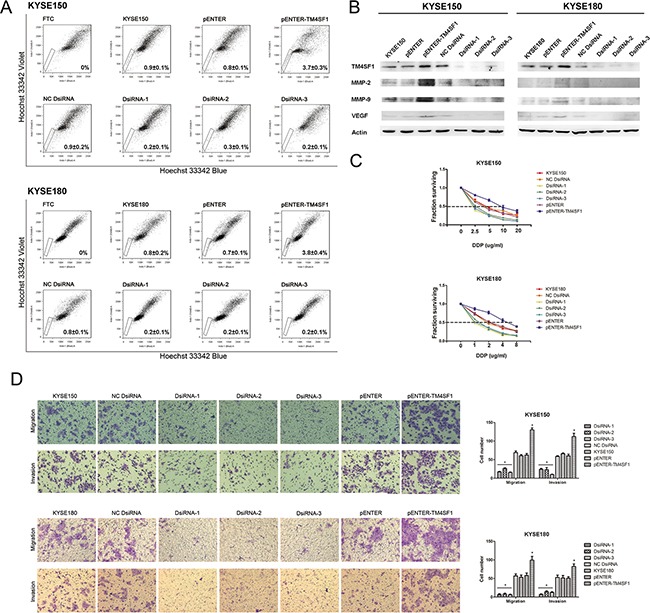
The effect of TM4SF1 expression on esophageal cancer stem-like cells, migration and invasion **A**. SP analysis of KYSE150 and KYSE180 cells after transfection or RNA interference. **B**. The expression of TM4SF1 and metastasis related proteins after transfection or RNA interference was measured by Western blot assay. **C**. IC50 of KYSE150 and KYSE180 cells after transfection or RNA interference to cisplatin by CCK8 assay (n=3, error bars indicate SD, P<0.05). **D**. Representative images from migration and invasion assay (n=3, error bars indicate SD, P<0.05).

To explore the function of TM4SF1, we over expressed TM4SF1 by transfecting pENTER-TM4SF1 vector and silenced TM4SF1 with three specific small interference RNAs (siRNAs) in KYSE150 and KYSE180 cells. Then, we performed SP analysis of KYSE150 and KYSE180 cell lines. The result showed that the SP fraction in the cells transfected with the pENTER-TM4SF1 vector was respectively significantly higher. Consistently, we found the SP fractions of the cells silenced of TM4SF1 expression was significantly lower (Figure 2A). The transfection efficiency was detected by Western blot assay (Figure 2B). These results demonstrated that the expression level of TM4SF1 could effectively regulate the SP phenotype of KYSE150 and KYSE180 cells.

### TM4SF1 promotes the chemotherapeutic resistant ability of esophageal cancer stem-like cells

As TM4SF1 regulated SP fraction, firstly we performed drug sensitivity assays by CCK8 with cisplatin which are commonly used of chemotherapy for ESCC. As shown in Figure 2C, after exposure to cisplatin, the viability of KYSE150 and KYSE180 cells transfected with the pENTER-TM4SF1 vector were markedly higher than that of parental KYSE150, KYSE180 cells or cells transfected with pENTER empty vector. And the viability of KYSE150 and KYSE180 cells transfected with siRNAs were markedly lower than that of KYSE150 and KYSE180 cells and transfected with negative control. In KYSE150 cells, the IC50 values were 4.604 for KYSE150, 4.638 for NC DsiRNA, 1.519 for DsiRNA-1, 2.260 for DsiRNA-2, 1.874 for DsiRNA-3, 3.859 for pENTER and 10.130 for pENTER-TM4SF1 separately. In KYSE180 cells, the IC50 values were 2.423 for KYSE180, 2.319 for NC DsiRNA, 0.856 for DsiRNA-1, 1.206 for DsiRNA-2, 1.037 for DsiRNA-3, 2.460 for pENTER and 5.263 for pENTER-TM4SF1 separately. As these cells did not differ in their growth rate (Supplementary Figure 1), these results suggested that the expression level of TM4SF1 in ESCC cells affected their chemotherapeutic drug resistance ability. Given the fact that we previously showed cancer stem-like cells of ESCC cells were more resistant to anticancer drugs [12], our results indicated that TM4SF1 could increase the resistant ability of esophageal cancer stem-like cells.

### TM4SF1 promotes migration and invasion in ESCC cells

In order to investigate whether TM4SF1 function in the progress of ESCC cells invasion and migration, we next performed cell migration and invasion transwell assays. The results showed that overexpression of TM4SF1 significantly enhanced migration and invasion of KYSE150 and KYSE180 cells compared with their corresponding control cells. On the contrary, silencing of TM4SF1 inhibited cell migration and invasion (Figure 2D). We also measured the expression levels of MMP-2, MMP-9 and VEGF which was known as the downstream proteins of TM4SF1 and involved in migration and invasion. We found that the expression of MMP-2, MMP-9 and VEGF was altered with the expression of TM4SF1 (Figure 2B). Taken together, these results support the notion that TM4SF1 plays an important role in ESCC cells migration and invasion.

### TM4SF1 promotes clonogencity and tumorigenic ability of ESCC cells

**Figure 3:**
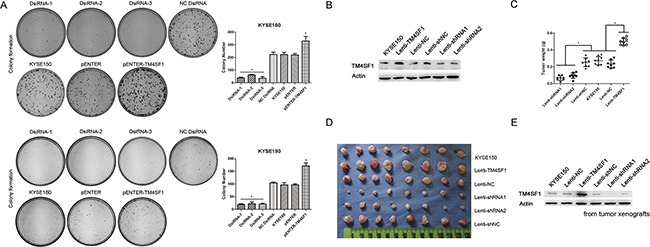
The effect of TM4SF1 expression on carcinogenicity **A**. Representative images from colony formation assay of KYSE150 and KYSE180 cells after transfection or RNA interference (n=3, error bars indicate SD, P<0.05). **B**. The expression of TM4SF1 after infection of KYSE150 cells with lentivirus was measured by Western blot assay. **C-D**. Tumor weights of lenti-TM4SF1, lenti-shRNA1, lenti-shRNA2 and control KYSE150, lenti-NC and lenti-shNC cells were plotted (n=8, error bars indicate SD, P<0.05). **E**. And the expression of TM4SF1 of tumor xengrafts was detected by Western blot assay.

To determine specifically whether TM4SF1 promotes clonogencity potential, we did the plate colony formation assay *in vitro*. We observed that the colony formation ability of cells were associated with the expression level of TM4SF1 (Figure 3A). Furthermore, in order to evaluate tumorigenic ability *in vivo*, we overexpressed and silenced TM4SF1 in KYSE150 cells using lentivirus-mediated transduction. The TM4SF1 expression was measured by Western blot assay (Figure 3B). Then, we injected the cells into nude mice, respectively. Four weeks after injection, weight measurement showed that tumor xenografts from lenti-TM4SF1 cells were much heavier than those from KYSE150 and lenti-NC cells. Similarly, tumor xenografts from lenti-shRNA1, lenti-shRNA2 cells were much lighter than those from KYSE150 and lenti-shNC cells (Figure 3C and 3D). In addition, we determined the TM4SF1 expression level of the tumors by Western blot assay. Consistently, the result showed that the change of TM4SF1 expression level was almost the same as that of cell lines’ (Figure 3E). These results indicated that the expression level of TM4SF1 in ESCC cells promotes the proliferation both *in vitro* and *in vivo*.

### TM4SF1 is a direct target of miR-141

**Figure 4:**
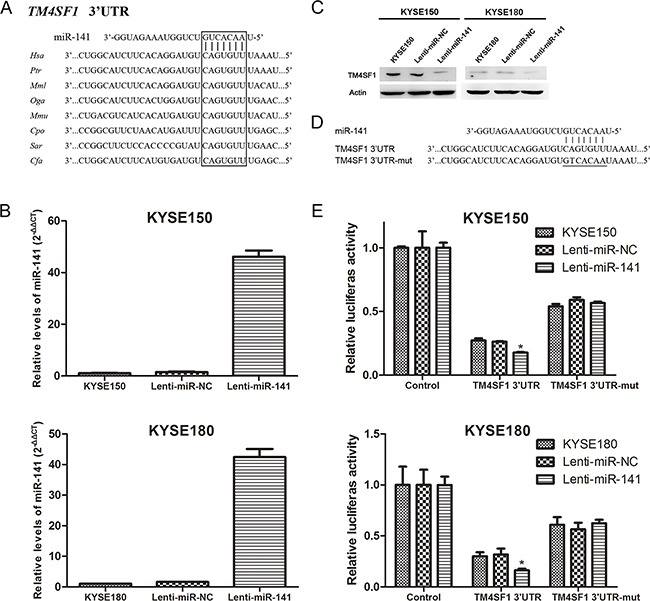
TM4SF1 is a direct target of miR-141 **A**. An miR-141 target site resides at +157 to +163 of the TM4SF1 3′UTR and is highly conserved in different species. **B**. After infection of cells with indicated lentivirus, miR-141 expression level was measured by real-time PCR and **C**. the expression of TM4SF1 mesured by Western blot after KYSE150 and KYSE180 cells infection with lentivirus. **D**. Diagram of luciferase reporter plasmids: plasmid with TM4SF1 3′UTR insert (pIS0-TM4SF1-3′UTR) and plasmid with a mutant TM4SF1 3′UTR (pIS0-TM4SF1-3′UTRmut) that carried a substitution of seven nucleotides within the miR-141 binding site. **E**. Luciferase acitivity assay demonstrates a direct targeting of the 3′UTR of TM4SF1 by miR-141. Cells were transfected with plasmids pIS0-TM4SF1-3′UTR or pIS0-TM4SF1-3′UTRmut. pRL-SV40 was used for normalization of transfection efficiency (n=3, error bars indicate SD, P<0.05).

The inverse correlation observed between miR-141 and TM4SF1 in expression suggested that TM4SF1 might be a direct target gene of miR-141. Therefore, we used Target Scan 6.0 to predict targets of miR-141 and found that the 3′UTR of TM4SF1 mRNA that matched perfectly to miR-141's 5′ seed sequence. Shown in Figure 4A is the miR-141 binding site at +157 to +163 nucleotides of TM4SF1 3′UTR. Comparing the sequence for interspecies homology, we found the miR-141 target sequence of TM4SF1 3′UTR is highly conserved among different species. We overexpressed miR-141 in KYSE150 and KYSE180 cells using lentivirus-mediated transduction. Compared with control cells, miR-141 expression was obviously increased in lenti-miR-141 cells (Figure 4B). Then, we found protein expression levels of TM4SF1 were significantly down-regulated in lenti-miR-141 cells (Figure 4C). Next, we generated TM4SF1-UTR and TM4SF1-UTRmut plasmids as the luciferase reporter and control, respectively (Figure 4D). The luciferase assay showed that, transfect of TM4SF1-UTR plasmid caused a decrease in the luciferase activity in lenti-miR-141 cells compared with controls, whereas mutation of the TM4SF1 3′UTR binding site weakened miR-141 mediated repression of the luciferase activity under the same conditions (Figure 4E). We also transfeced KYSE150 and KYSE180 with miR-141 mimics which overexpress miR-141. The results of transient transfection were consistent with the lentivirus transduction (Supplementary Figure 2). These results suggested that miR-141 directly targets TM4SF1 via the binding site in its 3′UTR region.

### The expression of miR-141 in human ESCC

**Figure 5:**
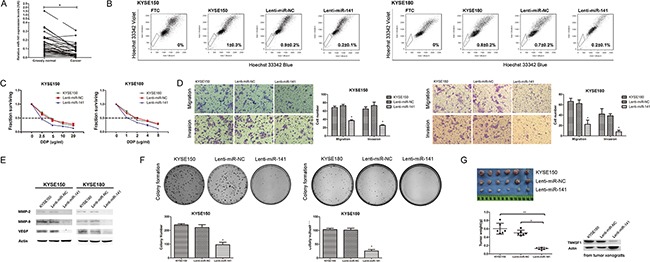
The effect of miR-141 overexpression on esophageal cancer stem-like cells **A**. The expression of miR-141 in human ESCC was measured by real-time PCR (n=36, *P<0.05). **B**. SP analysis of KYSE150, KYSE180 and their infection cells. **C**. IC50 of KYSE150, KYSE180 and their infection cells to cisplatin by CCK8 assay (n=3, error bars indicate SD, P<0.05). **D**. Representative images from migration and invasion assay (n=3, error bars indicate SD, P<0.05). **E**. The expression of metastasis and invasion related proteins after infection was measured by Western blot assay. **F**. Representative images from colony formation assay (n=3, error bars indicate SD, P<0.05). **G**. Tumor weights of KYSE150 cells and its infection cells (lenti-miR-NC and lenti-miR-141 cells) were plotted. And the expression of TM4SF1 of tumor xengrafts was detected by Western blot assay (n=6, error bars indicate SD, P<0.05).

In order to investigate the role of miR-141 in ESCC, we evaluated miR-141 expression by real-time RT-PCR in 36 couples of tissue specimens (tumor and normal tissue) of primary human ESCC. As shown in Figure 5A, we found the expression of miR-141 was significantly down-regulated in tumor than normal tissue of ESCC. This result suggested that miR-141 may play an important role in ESCC.

### MiR-141 regulates the SP phenotype

As we found that the expression of miR-141 was down regulated in SP cells, the results suggested that miR-141 might be involved in regulating esophageal cancer stem-like cells. Therefore we performed SP analysis. The results showed that the percentage of cells in the SP fraction of miR-141 overexpressed cells was significantly lower than that of its parental cells or lenti-miR-NC cells (Figure 5B). This result indicated that overexpression of miR-141 could effective regulate the SP phenotype of KYSE150 and KYSE180 cells.

### MiR-141 inhibits the chemotherapeutic resistant ability of esophageal cancer stem-like cells

To explore the function of miR-141, we performed drug sensitivity assays. After exposure to cisplatin, the viability of KYSE150 or lenti-miR-NC cells were markedly higher than that of lenti-miR-141 cells (Figure 5C). In KYSE150 cells, the IC50 values were 4.729 for KYSE150, 4.364 for lenti-miR-NC and 1.685 for lenti-miR-141. In KYSE180 cells, the IC50 values were 2.199 for KYSE180, 2.297 for lenti-miR-NCand 1.041 for lenti-miR-141. As these cells did not differ in their growth rate (Supplementary Figure 3), lenti-miR-141 cells were less resistant to cisplatin suggested that overexpression of miR-141 in ESCC cells affected their chemotherapeutic drug resistance.

### MiR-141 suppresses migration and invasion in ESCC cells

We next performed cell migration and invasion transwell assays. The results showed that overexpression of miR-141 significantly weaken migration and invasion compared with control cells (Figure 5D). We found that the expression of MMP-2, MMP-9 and VEGF was decreased after miR-141 overexpressed (Figure 5E). These results support the notion that miR-141 regulates TM4SF1 plays an important role in ESCC cells migration and invasion.

### MiR-141 inhibits clonogencity and tumorigenic ability of ESCC cells

To determine specifically whether miR-141 represses proliferative potential of ESCC cells, we tested the tumorigenic ability of these cells both *in vitro* and *in vivo*. We observed that colonies formed by lenti-miR-141 cells were smaller in size and less in number of plate colony formation assay (Figure 5F). Furthermore, tumorigenic ability was evaluated by injecting KYSE150, lenti- miR-NC or lenti-miR-141 cells into nude mice, respectively. Four weeks after injection, tumor xenografts were collected and detected of TM4SF1 expression level. As shown in Figure 5G, TM4SF1 was down-regulted in lenti-miR-141 cells, and weight measurement showed that tumor xenografts from lenti-miR-141 cells were much lighter than those from KYSE150 and lenti-miR-NC. These results indicated that overexpression of miR-141 in ESCC cells inhibits the carcinogenicity both *in vitro* and *in vivo*.

### MiR-141 regulates the esophageal cancer stem-like cells by suppressing TM4SF1

**Figure 6:**
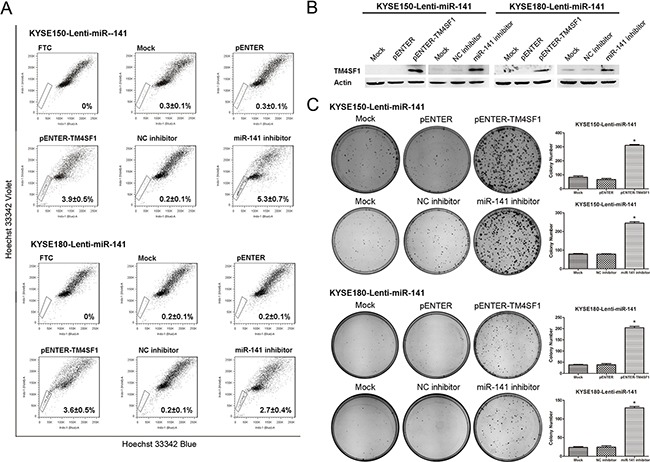
The effect of rescued TM4SF1 expression on esophageal cancer stem-like cells **A**. SP analysis of cells after transfection or RNA interference of KYSE150-Lenti-miR-141 and KYSE180-Lenti-miR-141 cells. **B**. The expression of TM4SF1 after transfection or RNA interference was measured by Western blot assay. **C**. Representative images from colony formation assay (n=3, error bars indicate SD, P<0.05).

To investigate the critical role of miR-141-TM4SF1 regulation in esophageal cancer stem-like cells in detail, we transfected pENTER-TM4SF1 plasmid and miR-141 inhibitor into the lenti-miR-141 cells to rescue the TM4SF1 expression. As shown in Figure 6B, ecotopic expression of TM4SF1 in lenti-miR-141 cells was successfully restored TM4SF1 protein expression. SP analysis indicated that the SP fraction was increased after TM4SF1 overexpression and miR-141 ablated in lenti-miR-141 cells (Figure 6A). Consistently, TM4SF1 rescued cells restored their clonogencity ability in plate colony formation assay (Figure 6C). These results indicated that TM4SF1 functioned as a stem renewal factor to be a key regulator factor in the maintenance of cancer stem-like cells and miR-141 regulated the esophageal cancer stem-like cells by suppressing TM4SF1.

## DISCUSSION

The Cancer stem-like cells hypothesis suggests that tumors are initiated and maintained by a minority subpopulation of cells that have the ability to self-renew and to generate the more differentiated progeny [34]. In recent years, the cancer stem cell model of tumorigenesis has received increasing attention. However, only a few studies have focused on the cancer stem-like cells of ESCC. The data presented here, coupled with those from our earlier report [11–13], demonstrate TM4SF1 and miR-141 could be a potential marker of cancer stem-like cells of ESCC. We found the expressions of TM4SF1 mRNA in cancerous and adjacent tissue of ESCC have no significant difference (Supplementary Figure 4). It is possible because esophageal cancer evolved from polyclonal origin cells [35, 36]. The reported incidence of esophageal patients with multiple carcinogenic lesions was 28.8%. The severe dysplasia cells were spread over 2/3 of the esophageal epithelial cell layer without breaking through the underlying membrane. The adjacent tissue (grossly) looks like normal, but multiple carcinogenic lesions, atypical hyperplasia, severe dysplasia, carcinoma in situ, or invasive carcinoma are presented in adjacent tissues of esophageal cancer analyzed by large pathological sections [37]. Therefore, in this study, we may not found the different expression of TM4SF1 between cancerous and adjacent tissue of ESCC. Another possibility is that the expression changes of TM4SF1 are only existed in cancer stem-like cells of ESCC. Then, we detected the expression of TM4SF1 and found it was increased in SP cells.

TM4SF1 has been reported to be associated with many tumors invasion and metastasis [38, 39], but has not been reported in esophageal cancer. The results presented in this study showed that TM4SF1 overexpression significantly enhanced KYSE150 and KYSE180 cells invasion and migration. TM4SF1 was found promote colony formation and carcinogenetic ability *in vivo* and *vitro*. Also, TM4SF1 increased the resistance to cisplatin of KYSE150 cells. Taken together, we can come to the conclusion that TM4SF1 could be a candidate surface protein marker that could discriminate cancer stem-like cells from ESCC cells, and could promote the ability to self-renew by increasing the number of cancer stem-like cells.

MiR-141 is a member of the miR-200 family, and is reported to be the potential biomarker of various diseases, including hepatocellular carcinoma [40], colorectal cancer [41]. However, the role of miR-141 in the development of ESCC remains unknown. As miR-141 and TM4SF1 were inversely expressed in SP cells, we explored TM4SF1 is a direct target gene of miR-141 and miR-141 could contribute to the self-renewal of esophageal cancer stem-like cells by suppressing TM4SF1. In addition, we detected the expression level of miR-200a, a critical member of miR-200 family, in SP and NSP cells of KYSE150 and KYSE180 cells. The result showed that miR-200a expression was also up-regulated in NSP cells (Supplementary Figure 5). It seemed that the effects we observed in this study are due to the combinational effects of miR-200 family members. But we found the up-regulation ratio (NSP/SP) of miR-141 expression was dramatically higher than miR-200a. And the expression of miR-141 was also higher than miR-200a in KYSE150 and KYSE180 cells. It is indicated that miR-141 may play a more important role than miR-200a in SP cells of ESCC.

In summary, we demonstrated that TM4SF1 was a direct target of miR-141. Regulation of TM4SF1 by miR-141 played an important role in controlling the cell proliferation and self-renewal of esophageal cancer stem-like cells. Thus, our results provide compelling evidence that miR-141 and TM4SF1 could be a potential target of the eliminating cancer stem-like cells in ESCC and might promote the development of new therapeutic strategies and efficient drugs to target ESCC stem-like cells.

## MATERIALS AND METHODS

### Ethics statement

Investigation has been conducted in accordance with the ethical standards and according to the Declaration of Helsinki and according to national and international guidelines and has been approved by the the ethics committees of Chinese Academy of Medical Sciences, Cancer hospital review board.

### ESCC tissues specimens

36 paired tissue specimens, tumors and adjacent non-tumor tissues of primary human ESCC were obtained from patients undergoing surgical resection for esophageal cancer. All of the tissues were obtained at the time of surgery and immediately stored in liquid nitrogen until use.

### Cell culture

The ESCC cell lines KYSE150 and KYSE180 were gifts from Dr.Y.Shimada, and maintained in RPMI 1640 supplemented with 10% FBS at 37°C and 5% CO_2_.

### Analyzing and sorting of cell lines by FACS

The procedure for SP analysis is based on procedures previously described [11–13]. Cells were sorted using dual-wavelength analysis with BD FACS Vantage SE (Becton, Dickinson and Company, Franklin Lakes, NJ). The SP gate was defined as the diminished area on the dot plot in the presence of fumitremorgin C (FTC).

### RNA isolation and real-time PCR

Total RNA was extracted with Trizol reagent (Invitrogen, Carlsbad, CA, USA). The expression of TM4SF1 were carried out according to the protocol of SYRB Premix Ex Taq^TM^ Perfect Real Time system (Takara, Dalian, China). The expression of miR-141 were measured by a two-step TaqMan assay (Applied Biosystems). GAPDH or U6 was used as the internal control. Fold changes in TM4SF1 and miR-141 expression were calculated using the 2-ΔΔCt method [42]. The primers used are listed in Supplementary Table 1.

### Western blot analysis

Cultured cells were harvested and total cellular extracts were prepared by cell lysis buffer, and Western blot analysis was performed as previously described [13]. The primary antibodies of TM4SF1 (Abcam, Cambridge, MA, USA) and β-actin (Sigma) were used.

### Trasfection and infection

Cell transfections were performed using LipofectAMINE 2000 (Invitrogen). For infection, lentiviruses were generated by triple transfection of 80% confluent HEK293T cells with modified plasmids and harvested after 3 days. KYSE150 and KYSE180 cell lines were infected at MOI 100.

### CCK8 and sensitivity to chemotherapeutic reagents assays

The assay was performed essentially as protocols of CCK8 assay kit (Diojindo Molecular Technologies, Tokyo, Japan). Cisplatin was added to the medium as the representative chemotherapeutic agents.

### Migration and invasion assays

Migration and matrigel invasion assays were analyzed using a standard Boyden Chamber protocol. Briefly, 2 × 10^5^ cells in 1640 without FBS were loaded into the upper chamber, serum in the complete medium as the chemoattractant in the lower chamber. Cells were allowed to migrate towards complete media for 24 hours. Non-migrated cells were removed and nuclei of migrating cells were stained.

### Plate colony formation assay

Suspensions of logarithmically growing cells at a density of 500 cells per well were seeded in the 60×15mm dishes for plate colony formation assay. After 2 weeks, colonies were stained with crystal violet staining.

### DNA constructs and RNA interference

The TM4SF1 vector (pENTER-TM4SF1, Cat: CH891913, RefSeq: NM014220) and empty vector pENTER were purchased from ViGene CO., Ltd., (Shandong,China). TM4SF1 small interference RNAs (siRNAs) and negative control were synthesized by Genechem Co., Ltd., (Shanghai, China). For construction of lenti-TM4SF1, its open reading fragment of cDNA was cloned to the lentiviral vector PLVX-IRES-Neo (Clontech). Two shRNA (shRNA1,shRNA2) constructs targeting TM4SF1 were cloned to lentiviral vector pSIH1-H1-Puro (System Biosciences). Lenti-NC or Lenti-shNC was generated as a control. For construction of human TM4SF1 3′UTR reporter plasmid, nucleotides + 844 to + 1691 of human TM4SF1 cDNA were cloned into the pIS0 luciferase plasmid. Mutant construct of TM4SF1 3′UTR, named TM4SF1 3′UTR-mut, which carried a substitution of seven nucleotides within the core binding site of TM4SF13′-UTR, was carried out using MutanBEST Kit (Takara, Dalian, China). The primers and sequences used are listed in Supplementary Table 1.

### Lucifer's reporter assay

The KYSE150 and KYSE180 cells in 96-well plate were transfected with firefly luciferase reporter gene plasmid (100ng). Transfection efficiency was standardized by cotransfection with pRL-SV40 (1.0ng). The luciferase activity was determined using the Luciferase Assay system (Promega). The fold increase was calculated by defining the activity of the empty pIS0 plasmid as 1.

### Xenograft assay in mice

All experiments were carried out in accordance with procedures approved by the Animal Care and Use Committee of CICAMS. The freshly prepared (1×10^6^/each) cells were injected subcutaneously into the left axillary fossa of female nude mice (4 weeks old). The mice were monitored twice a week for palpable tumor formation and were killed at 4 weeks after transplantation to determine tumor formation.

### Statistical analysis

Statistical software SPSS13.0 was used in data processing and for analyzing the significance among groups. P <0.05 was considered statistically significant.

## SUPPLEMENTARY FIGURES AND TABLE



## ABBREVIATIONS

SP: side population
NSP: none side population
ESCC: esophageal squamous cell carcinoma
FTC: fumitremorgin C
