# Supplementary material for: TM4SF1 promotes the self-renewal of esophageal cancer stem-like cells and is regulated by miR-141
Source: Oncotarget. 2016 Dec 10;8(12):19274–84. doi: 10.18632/oncotarget.13866 (PMC5386683; doi:10.18632/oncotarget.13866)
Supplement: Supplementary file 1 [file oncotarget-08-19274-s001.pdf]

TM4SF1 promotes the self-renewal of esophageal cancer stem-like cells and is regulated by miR-141

SUPPLEMENTARY FIGURES AND TABLE

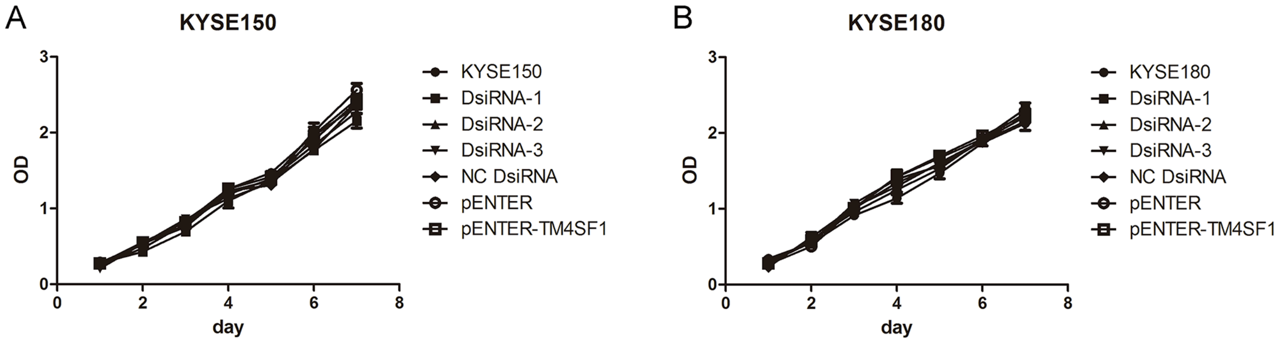

Supplementary Figure 1: Growth curves.

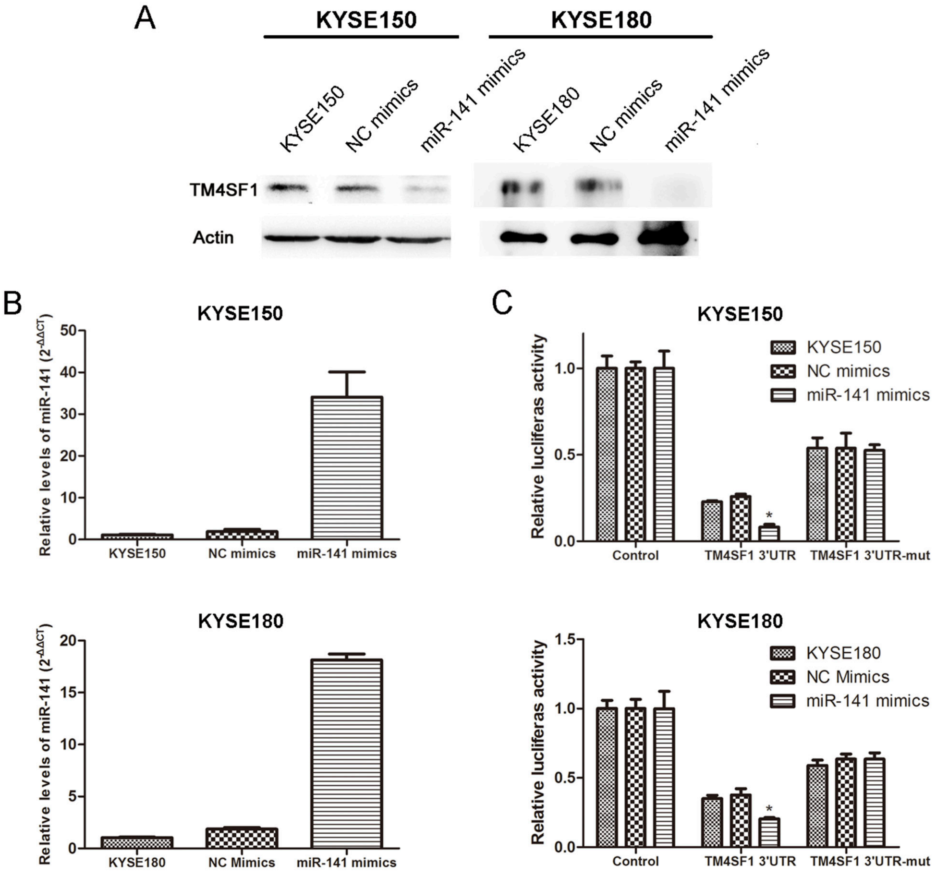

Supplementary Figure 2: Western blot, real-time PCR and luciferase assays for miR-141 mimics.

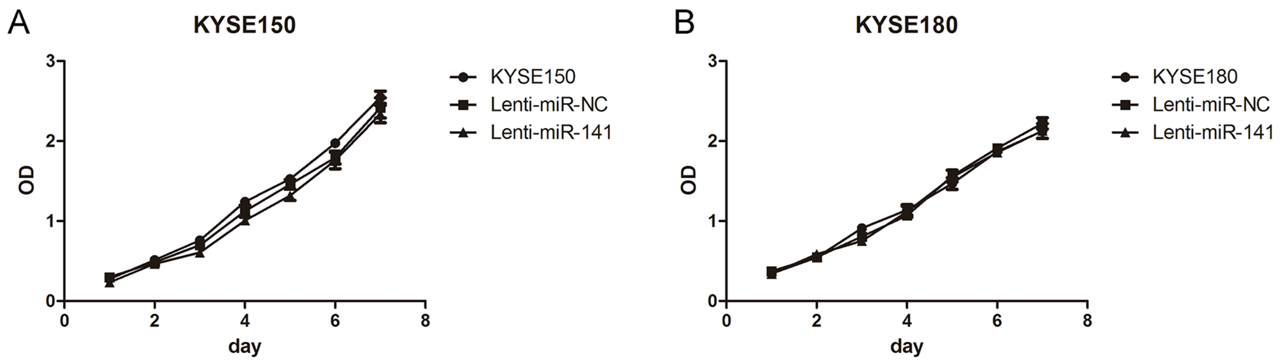

Supplementary Figure 3: Growth curves.

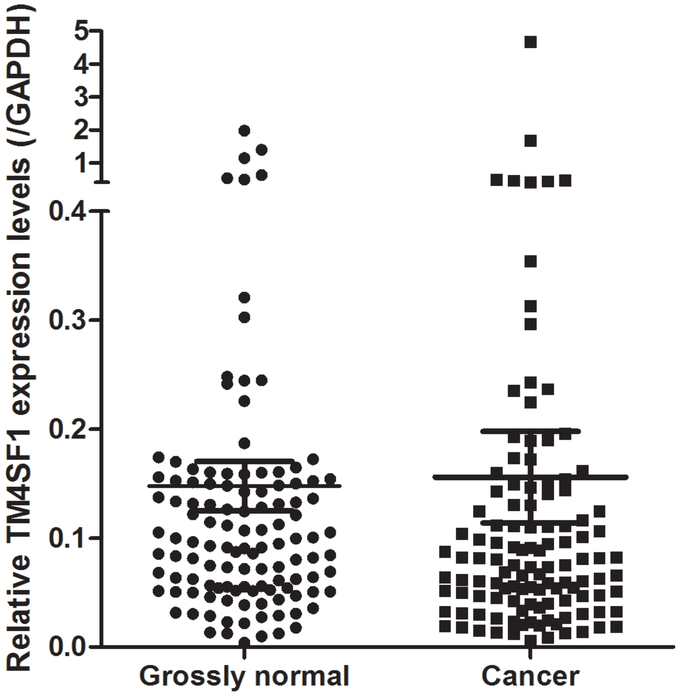

Supplementary Figure 4: The RNA expression of TM4SF1 in human ESCC was measured by real-time PCR.

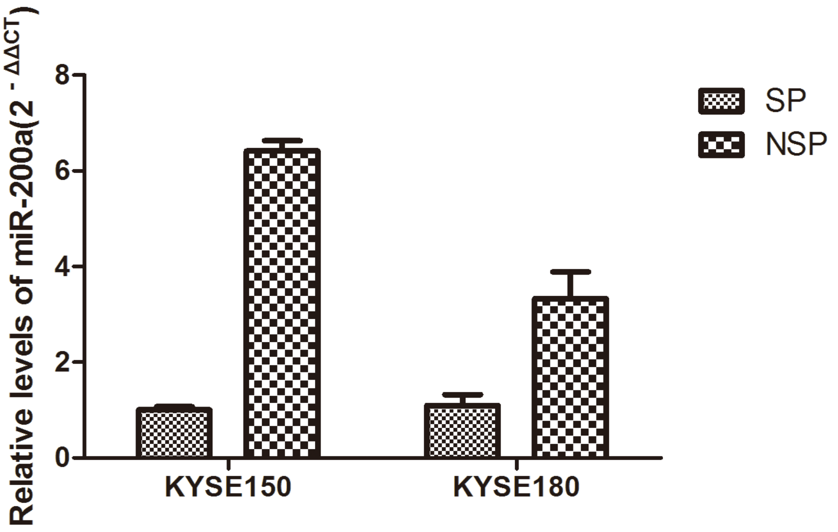

Supplementary Figure 5: In SP and non-SP cells of KYSE150 and KYSE180 cell lines, miR-200a expression level was measured by real-time PCR.

Supplementary Table 1: Primer or probe sequences

Name	Primers/Sequences
TM4SF1-rt-f	5’-CAGCCCTTGGCTTAGCAGA-3’
TM4SF1-rt-r	5’-CCACAATGCTTGGGTTCA-3’
GAPDH-rt-f	5’-GAGTCAACGGATTTGGTCGT-3’
GAPDH-rt-r	5’-GACAAGCTTCCCGTTCTCAG-3’
Lenti-TM4SF1-XbaI	5’-GTCTAGAATGTGCTATGGGAAGTGTGC-3’
Lenti-TM4SF1-BamHI	5’-GTCTAGAATGTGCTATGGGAAGTGTGC-3’
Lenti-TM4SF1-shRNA1	5’-GCGATGCTTTCTTCTGTATTT-3’
Lenti-TM4SF1-shRNA2	5’-GCTCTCACCAACAGCAATATT-3’
TM4SF1UTR-SacI	5’-GAGCTCAAGAACCAACCCAGGACAG-3’
TM4SF1UTR-XbaI	5’-TCTAGAAACATGGTAGTGAATACTTTA-3’
TM4SF1UTRmut-f	5’-TGTCACAATAAATTTAGTAAACTTCTT-3’
TM4SF1UTRmut-r	5’-CATCCTGTGAAGATGCCAGTC-3’
TM4SF1-homo-497	5’-GCGAUGCUUUCUUCUGUAUtt-3’(siRNA1)
TM4SF1-homo-733	5’-GGCUCUUGGUGGAAUUGAAtt-3’(siRNA2)
TM4SF1-homo-813	5’-GCUCUCACCAACAGCAAUAtt-3’(siRNA3)
MiR-141 inhibitor	5’-CCAUCUUUACCAGACAGUGUUA-3’
